# Alcohol Drinking in University Students Matters for Their Self-Rated Health Status: A Cross-sectional Study in Three European Countries

**DOI:** 10.3389/fpubh.2016.00210

**Published:** 2016-09-27

**Authors:** Rafael T. Mikolajczyk, Rene Sebena, Julia Warich, Vihra Naydenova, Urszula Dudziak, Olga Orosova

**Affiliations:** ^1^Department of Epidemiology of Infectious Diseases, Hannover Medical School, Hannover, Germany; ^2^Department of Epidemiology, Helmholtz-Centre for Infection Research, Braunschweig, Germany; ^3^Department of Psychology, Faculty of Arts, PJ Šafárik University, Košice, Slovakia; ^4^Department of Public Health Medicine, School of Public Health, Bielefeld University, Bielefeld, Germany; ^5^Faculty of Philosophy, Sofia University, Sofia, Bulgaria; ^6^Institute of Family Sciences, Catholic University of Lublin, Lublin, Poland

**Keywords:** self-rated health, problem drinking, emerging adulthood, cross-country comparison, CNSHS

## Abstract

**Background:**

Alcohol drinking was linked to self-rated health in different populations, but the observed association was inconsistent. We studied the association among university students across three European countries with different patterns of drinking.

**Methods:**

We analyzed data from three universities, one from each country: Germany (beer dominant), Bulgaria (wine dominant), and Poland (unclassified among youths, spirits dominant in adults) (*N* = 2103). Frequency of drinking and problem drinking (≥2 positive responses on CAGE-scale), on the one side, and self-rated health, caring for one’s own health, and worsening of health since the last year, on the other side, were assessed by means of self-administered questionnaire. The association between alcohol- (independent) and health-related (dependent) variables was evaluated by means of logistic regression, adjusting for country and sex.

**Results:**

Poor self-rated health and worsened health since previous year were associated with problem drinking {odds ratio 1.82 [95% confidence interval (CI) 1.21–2.73] and 1.61 (95% CI 1.17–2.21), respectively}, but not with a higher frequency of drinking. In contrast, not caring for one’s own health was associated with frequent drinking [1.40 (95% CI 1.10–1.78)], but not with problem drinking [1.25 (95% CI 0.95–1.63)]. The results were consistent across the studied countries and for both sexes.

**Conclusion:**

The health status of university students was associated with problem drinking. A high frequency of drinking was associated with the lack of care of own health, but it was not associated with current health status. These associations were independent of the predominant pattern of drinking across the studied countries.

## Introduction

Excessive alcohol consumption is one of the major public health challenges in Europe. Globally, alcohol use is the third leading risk factor for poor health and causes an estimated 2.5 million deaths per year. Alcohol-related problems are diverse and have a strong social dimension; a significant proportion of problems occur among the young (1). Abusive or harmful alcohol consumption is related to mental and physical health problems, which include risky behaviors, such as violence, road accidents, and social isolation, for example. These are especially relevant for countries of Central and Eastern Europe, often referred to as a homogenous group, although, in reality, there exists a variety of populations, cultures, and economics and therefore a variety of drinking patterns in these regions (2). The regional heterogeneity further increased by different trajectories of social and economic transition in those countries in the last 20 years (3, 4). A drastic increase in problems related to alcohol drinking among young people has been observed in Poland in recent years, for instance (5). The need for comprehensive research on the alcohol use and drinking patterns among adolescents and young adults in Europe has been repeatedly articulated (6).

Alcohol drinking typically begins in high school and the first relevant experience of alcohol consumption in adolescence often manifest in young adulthood. Alcohol drinking escalates upon college entry, being intensified by the university environment. The use of alcohol can gradually progress into abuse and even dependence later in life; just as it is possible that the consumption gradually decreases during college years, and then drops off following college (7, 8). A common pattern of drinking among students is the so-called heavy episodic or binge drinking, defined as drinking at least five drinks on one occasion for men or at least four drinks on one occasion for women (9). University students do not only have a unique drinking pattern in general but also have a higher alcohol consumption than other age groups and even within the same age group in comparison to non-students (10, 11). Therefore, they are at a higher risk of developing alcohol use disorders than non-college students. The effect of heavy episodic drinking has been well documented for student populations in the U.S. (9, 10, 12), but there is less comparable European research.

In comparison to the U.S., Europe is not only more diverse in its cultural and regional aspects but also alcohol-related patterns vary (13). Meanwhile, the traditional drinking patterns of wine in the south (the Mediterranean drinking pattern), beer in Central Europe (Central European drinking pattern), and spirits in the Nordic countries (Northern European drinking pattern) are not up-to-date anymore, especially among young people (14, 15).

As examples of the European drinking cultures, three countries (Germany, Poland, and Bulgaria) were chosen for this study. Germany has been chosen as a traditional beer drinking country (11.6–12.9 lg of alcohol per capita), Poland for vodka (8.7 lg alcohol of per capita), and Bulgaria for wine (6.4–7.1 lg of alcohol per capita) (5, 14, 15). The World Health Organization has defined Germany as a beer and Bulgaria as a wine drinking country; it has not classified Poland to any specific preference of beverage, taking into account the high consumption of beer in the Polish population. However, in all three nations, the converging of drinking culture into the Central European drinking pattern, mainly beer as beverage preference, has been observed over time and is well documented – especially for students (13, 15).

Binge drinking is a common risky behavior among college students and has been associated with suboptimal self-rated health (7, 10, 16, 17). If a person’s perception of his/her self-rated overall health status is influenced by the use of alcohol, type of beverage, and the individual drinking pattern has been controversially discussed in literature (17–21). Recently, the association between self-rated health and the type of alcohol beverage consumed has been investigated by Valencia-Martin et al. (19), with the conclusion that wine and beer drinkers are less likely to binge drink than spirits drinkers and the commonly invoked a J-shaped association of self-rated health and the level of alcohol consumption was misinterpreted. Consequently, self-rated health is mainly affected by the drinking pattern and not the level of alcohol consumption. Frequent binge drinking was associated with a significantly worse health-related quality of life and mental distress among U.S. adults (≥ 18 years) (21). Similarly, episodic heavy alcohol consumption was strongly associated with poor self-rated health according to the findings in a representative sample of American adults (20). Among students, the relationship between alcohol consumption and mental health was studied extensively [e.g., Ref. (22)], but the potential effects on physical health were understudied.

The aim of this study was to determine whether the effect of alcohol has on the self-rated health differs in younger populations across countries with different predominant drinking cultures. Therefore, the analysis examines the associations between frequent drinking and problem drinking and self-rated health in a sample of university students in three European countries.

## Materials and Methods

### Sample

The dataset used in this study was collected as a part of the Cross-National Student Health Study (CNSHS), which was conducted as an open collaboration of universities from several European and Mediterranean countries (23). In the three countries, Germany, Bulgaria, and Poland, the survey was conducted in 2005 at one university in each participating country: Bielefeld University in Bielefeld, Germany; the Catholic University of Lublin, Lublin, Poland; the University of Sofia, Sofia, Bulgaria. The study applied a self-administered multi-theme health survey, including questions on alcohol use and health behaviors. The original questionnaire was developed in German and translated into Polish and Bulgarian by two independent translators. The two versions were compared, and their disagreements resolved in discussion with native speakers involved in the research team. The questionnaire was distributed to first-year students during regular classes in the final part of selected courses and collected immediately after being completed by the students. At each university, courses were selected to achieve nearly equal representation of social sciences, natural sciences, and law and economy. The response rate was 85% in the German sample and above 95% in the Polish and Bulgarian samples. The final sample included 2103 students. In order to increase the accuracy of self-reports, students were assured that their answers would remain confidential, and data protection was observed at all times.

### Measures

#### Alcohol-Related Variables

The frequency of alcohol consumption was measured using the following question: “Over the past 3 months how often have you drunk alcohol, for example, beer?” The possible answers were: “a few times per day,” “every day,” “a few times per week,” “once per week,” and “less than once per week or never.” For the purpose of this analysis, these five categories were recoded into three categories: frequent drinking (“a few times per day,” “every day,” and “a few times per week”), occasional drinking (“once a week”), and rare (“less than once per week or never”). Problem drinking was measured using the CAGE test. The CAGE test (24) is a brief screening instrument consisting of four short questions (Have you ever felt you should cut down on your drinking? Have people annoyed you by criticizing your drinking? Have you ever felt bad or guilty about your drinking? Have you ever had a drink in the morning to get rid of a hangover?). Each question is answered either with “yes” or “no.” Two or three affirmative answers suggest problem drinking, while four positive responses allow serious suspicion of alcohol dependence.

#### Health-Related Variables

To assess health status of the students, we applied the generic scale for self-rated health “How would you rate your health in general?” with a five-point response scale “excellent,” “very good,” “good,” “fair,” and “poor” (25). Additionally, we asked about health changes in the last year “Compared with the past year how would you describe your health condition?” with five possible responses: “now much better than a year ago,” “now a bit better than a year ago,” “almost the same as a year ago,” “now worse a bit than a year ago,” and “now much worse than a year ago.” We also asked whether students care for their health (How well do you take care of your health?) with four possible responses “not at all,” “quite little,” “quite lot,” and “a lot.”

### Informed Consent and Ethical Permission

Participation in the study was voluntary and anonymous. Students were informed that by completing the questionnaire they were providing their informed consent to participate. They were also informed that they could terminate the participation at any point while filling out the questionnaire. The permission to conduct the study was granted by the participating institutions: Bielefeld University in Bielefeld (Germany), the Catholic University of Lublin, Lublin (Poland), the University of Sofia in Sofia (Bulgaria).

### Statistical Analysis

The analysis was stratified by country and sex for descriptive purposes. In order to investigate whether there are any differences across countries and by sex, a new variable was created with all country/sex combinations. The association between health-related and alcohol-related variables was studied in separate logistic regression models, with alcohol-related variables and sex/country variable as independent and health-related variables as dependent. The differential effects of country/sex combinations for the association between health- and alcohol-related variables were studied by assessing whether the interaction between the country/sex variable and alcohol-related variables was significant (*p* < 0.05).

## Results

Basic demographic information of the sample is provided in Table 1. First semester students in Bulgarian sample were the youngest, followed by students from Poland and from Germany. Among the three countries, students in Bulgaria reported the most frequent alcohol consumption and in Poland the least frequent (Table 2). Consistently, frequent alcohol consumption was more common among males than females in all three countries. Also, problem drinking measured by CAGE was more common among males than females. Across the studied countries, the differences in problem drinking were less pronounced than the differences in drinking frequency, with a very similar fraction of women reporting problem drinking in all three countries.

**Table 1:** **Basic demographic information about the sample by country**.

	Bulgaria	Germany	Poland
*N* respondents	701	770	572
Sex (%)			
Female	68.3	57.7	71.3
Male	31.7	42.3	28.7
Age category (%)			
<20 years	55.0	1.8	23.0
20–22 years	42.7	72.0	74.0
>22 years	2.3	26.2	3.0
Country of birth			
Same as country of study	97.6	95.9	98.7
Different as country of study	2.4	4.1	1.3

**Table 2:** **Alcohol- and health-related variables by country and sex**.

Country	Bulgaria		Germany		Poland		*p*-Value*
				
	Females	Males	Females	Males	Females	Males	
*N* respondents	*N* = 479	*N* = 222	*N* = 444	*N* = 326	*N* = 408	*N* = 164	
Alcohol consumption^b^	*N* = 477^a^	*N* = 221^a^	*N* = 431^a^	*N* = 319^a^	*N* = 399^a^	*N* = 159^a^	<0.001
Frequent (%)	28.1	46.2	18.1	41.1	10.0	20.1	
Occasional (%)	38.6	33.9	31.6	31.7	13.0	21.4	
Rare (%)	33.3	19.9	50.3	27.3	76.9	58.5	
Problem drinking^c^	*N* = 448^a^	*N* = 211^a^	*N* = 407^a^	*N* = 308^a^	*N* = 378^a^	*N* = 153^a^	<0.001
Yes (%)	9.6	22.7	9.3	26.3	8.5	19.0	
No (%)	90.4	77.3	90.7	73.7	91.5	81.0	
Self-rated health (current)^d^	*N* = 475^a^	*N* = 221^a^	*N* = 440^a^	*N* = 319^a^	*N* = 407^a^	*N* = 163^a^	<0.001
Good (%)	92.2	95.9	90.5	92.5	83.8	85.9	
Poor (%)	7.8	4.1	9.5	7.5	16.2	14.1	
Health compared to previous year^e^	*N* = 476^a^	*N* = 218^a^	*N* = 438^a^	*N* = 320^a^	*N* = 408^a^	*N* = 161^a^	<0.001
Stable or improved (%)	83.8	90.4	81.1	79.4	70.3	82.6	
Worsened (%)	16.2	9.6	18.9	20.6	29.7	17.4	
Caring for one’s health^f^	*N* = 476^a^	*N* = 216^a^	*N* = 442^a^	*N* = 318^a^	*N* = 406^a^	*N* = 159^a^	<0.001
Yes (%)	46.2	47.2	68.3	58.8	56.4	47.8	
No (%)	53.8	52.8	31.7	41.2	43.6	52.2	

*Chi-squared test across country/sex combinations.

*^a^Valid cases*.

^b^Frequent: “a few times per day,” “every day,” “a few times per week”; Occasional: “once per week”; Rare: “less than once per week and never.”

*^c^Problem drinking: yes 2 or more positive responses in CAGE (includes the category “at risk for alcoholism,” which requires 4 positive responses); No: abstainers or <2 positive responses in CAGE*.

*^d^Good: excellent, very good, good; Poor: fair, poor*.

*^e^Stable or improved: now much better than a year ago, now a bit better than a year ago, almost the same as a year ago; worsened: now worse a bit than a year ago, now much worse than a year ago*.

*^f^Yes: a lot, quite a lot; no: not at all, quite a little*.

Poor self-rated health was slightly more common in female students in all three countries and substantially more common in Poland than in the other countries. Between 10 and 30% reported a worsening of health during the last year. In Bulgaria and Poland, this fraction was higher among female than male students. In Poland and Germany, females reported higher levels of caring for one’s health than males, in Bulgaria, females’ and males’ responses were rather similar in this respect. Fractions of students responding that their care for their health were substantially larger in Germany than in the two remaining countries.

Neither poor self-rated health nor worsening of health since previous year was associated with frequent or occasional drinking (Table 3). However, persons who drank alcohol frequently were also 40% more likely to report not to care for their own health. On the contrary, there were strong associations between poor self-rated health and worsening of health since previous year and problem drinking (80% higher risk of poor health and 60% higher risk for worsening of health for students with problem drinking). The relationship between not caring for own health and problem drinking did not reach statistical significance. All these associations did not differ across countries and by sex (none of the interactions between the joint country and sex variable and alcohol-related variables was significant).

**Table 3:** **Association between health-related and alcohol-related variables (odds ratios from logistic regression with 95% confidence intervals, separate models for drinking frequency and problem drinking)**.

	Poor self-rated health	Worsened health since previous year	Not caring for own health
	
	OR (95% CI)^a^	OR (95% CI)^a^	OR (95% CI)^a^
**Drinking frequency**			
Frequent vs. no	1.15 (0.77–1.71)	1.08 (0.80–1.46)	1.40 (1.10–1.78)*
Occasional vs. no	1.17 (0.81–1.70)	0.93 (0.70–1.23)	1.20 (0.96–1.51)
**Problem drinking**			
Yes vs. no	1.82 (1.21–2.73)*	1.61 (1.17–2.21)*	1.25 (0.95–1.63)

*No interactions between country/sex and alcohol-related variables were significant at p < 0.05 level*.

*^a^Adjusted for all country/sex combinations*.

*p < 0.05.

## Discussion

This study described the prevalence of frequent alcohol consumption and the prevalence of problem drinking among university students from three European countries: Germany as a Western European country, Poland as Central European country, and Bulgaria as representative for a Southern European country. In agreement with previous studies undertaken in Europe (13, 14), the highest rates of frequent drinking were found in Bulgaria and Germany. There is a high level of alcohol consumption among Bulgarian students compared with the other countries. These results confirm the tendency shown in the other studies on alcohol consumption carried out in the last years among young Bulgarians, who are described as a high-risk group (26). Bulgaria has been faced with a lot of public health challenges in the transition period from communism to market economy, especially in the field of health and health behaviors of young people. On contrary, the results of contemporary studies comparing heavy alcohol consumption in Bulgaria with other central and eastern European countries show that in Bulgaria the proportion of heavy alcohol consumption among men is the lowest compared with all other countries from these regions (14). On the other hand, the lowest prevalence of frequent drinking was found in Poland. Alcohol consumption in Poland is characterized by a relatively low drinking frequency and a concentrated consumption of large quantities when drinking does occur (27). Ostaszewski and Pisarska (4) indicated the stabilization of youth alcohol use in Poland according to the Mokotów study 1988–2004 (the monitoring that was carried out from 2000 to 2004), but Lisicki (28) obtained results that documented independently from the type of the school, that as the years of studying pass, the percentage of university students who consume alcohol increases. Overall, we support the hypothesis of higher drinking frequencies in Southern (Bulgaria) and Western (Germany) European countries and a low drinking frequency in Poland.

Consistent with the previous studies (29, 30), frequent alcohol consumption as well as problem drinking was more common among males than females in all three countries. The common explanations are biological (31) and social–psychological (32). We also found slightly more common poor self-rated health in female students than in male students in all three countries. The U.S. National Health Interview Surveys throughout the 1990s also showed slightly higher percentages of women rating their health as fair or poor, compared with men. Interesting related finding was observed in studies assessing the association between self-rated health and mortality that report that while there are more men in the poorest health category, women are reporting more often poorer health (25, 33).

In relation to cross-national differences in the self-reported health, we found poor self-rated health substantially more often in Poland than in the other two countries. Another international comparative study (34) confirmed that university students from Poland self-rated their health status lower in comparison to students from Germany. The differences in health perception between Eastern and Western Europe might be due to a lower perception of control in Eastern European countries due to instabilities during political and economic transitions (35). These inequalities in health may be also linked to socio-economic and cultural differences among the European regions.

Main purpose of this study was to investigate the association between self-reported health and alcohol drinking. The strong association between poor self-rated health, worsening of health since previous year and problem drinking confirms the hypothesis that alcohol problem drinking can be defined as being strongly connected to worse health in the surveyed young people. Previous research exploring the association between alcohol consumption and self-rated poor health provided inconsistent and even contradictory findings. In a cross-sectional study covering persons aged 25–64 years in three areas of Finland, a “J-shaped” relation was observed between average alcohol consumption and self-rated poor health (36). Similar findings were observed in the Danish population in Copenhagen (37). In contrast, two other studies from the Netherlands and Spain showed that as alcohol consumption increases the frequency of ill-health decreases (38, 39). Also, a Japanese study reported a positive association between moderate alcohol use and good self-rated health (40). Our findings contribute to the clarification of this disagreement, as we were able to compare a simple measurement of drinking frequency with an instrument assessing problem drinking. While the current frequency of drinking itself was not associated in this young sample with health impairment, this was the case for problem drinking measured by CAGE. Since CAGE focuses more on long-term drinking habits, it is more likely that it will be associated with health effects, such as anxiety, depression, sleep problems, or other mental health or health problems. Excessive alcohol consumption over a period of time can begin to affect almost every system in the body. Interestingly, problem drinking was not associated with caring less for one’s health. Possibly, it means that persons who score high on CAGE realized that they have a problem with drinking and are concerned about their health, thus obscuring the originally negative association.

We did find neither poor self-rated health nor worsening of health since previous year being associated with frequent or occasional drinking in contrast to rare drinking. This lack of association did not differ with respect to investigated countries. Since frequency of drinking is such a broad measure, which does not assess the quantity of alcohol consumed, high reported frequency presents only a relatively small concern for health problems. Other possible explanation is that university students represent relative healthy population, not yet influenced by negative effects of excessive alcohol use or abuse. Negative effects on health may be relevant in later developmental stages. On the other hand, we found persons who drank alcohol frequently more likely not to care for the own health. Higher amounts of alcohol may be also connected with various maladaptive outcomes, alcohol-related problems, and self-neglecting behavior that can extend far into adulthood.

### Limitations and Strengths

The strength of the study is the use of a common questionnaire across countries with different drinking patterns and relatively large, homogenous sample. But, the study has also several limitations. With respect to alcohol, we did not ask about the kind of drinks in the studied sample but relied on the known patterns of drinking in the countries. We also assessed only three alcohol-related variables, instead of a more comprehensive appraisal. Similarly, we used short questions to assess health status rather than more comprehensive scales. The questions were included in a multitopic survey, and thus the number of specific variables was limited. The student sample from each country was not representative, and collected data were only self-reported. On the other side, given the high response rate, selection bias is unlikely to affect the findings of the study, and the advantage of self-reported questionnaire is that it unlikely introduces a strong social-expectation bias in the responses of participants. Both aspects support the internal validity of the study. Since the study was cross-sectional in nature, causal conclusions about the role of alcohol drinking cannot be derived. The nearly disjunct age distributions across the countries did not allow controlling for age in the analysis.

## Conclusion

Problem drinking is associated with poor self-rated health and worsening of health in university students from the studied countries. In contrast, more frequent drinking is associated with not caring for one’s health, while there is no association between problem drinking and caring for one’s health. These associations are similar for male and female students, despite the differences between both sexes in the prevalence of frequent drinking and problem drinking, on the one side, and in health status, on the other side. The associations between alcohol- and health-related variables were also independent of the dominant patterns of drinking of the studied countries.

## Author Contributions

RM developed the research question, conducted the analysis, and drafted parts of the manuscript. RS and JW conducted literature search and contributed to writing and analysis. VN, UD, and OO contributed to writing. RM, VN, UD, and OO conducted the original study.

## Conflict of Interest Statement

The authors declare that the research was conducted in the absence of any commercial or financial relationships that could be construed as a potential conflict of interest.
